# Attractive Gait Training: Applying Dynamical Systems Theory to the Improvement of Locomotor Performance Across the Lifespan

**DOI:** 10.3389/fphys.2018.01934

**Published:** 2019-01-18

**Authors:** Bas Van Hooren, Kenneth Meijer, Christopher McCrum

**Affiliations:** ^1^Department of Nutrition and Movement Sciences, NUTRIM School of Nutrition and Translational Research in Metabolism, Maastricht University Medical Centre+, Maastricht, Netherlands; ^2^Institute of Sport Studies, Fontys University of Applied Sciences, Eindhoven, Netherlands; ^3^Institute of Movement and Sport Gerontology, German Sport University Cologne, Cologne, Germany

**Keywords:** locomotion, gait, running, dynamic stability, injury prevention, falls prevention

## Introduction

Healthy adult humans can walk and run with ease, yet it takes years to develop stable and economical locomotion. This apparent ease is the result of multiple degrees of freedom at dozens of joints being controlled by hundreds of muscles, all recruited and activated with precise timing and frequency by the neuromotor system (Turvey, 1990; Pandy and Andriacchi, 2010; Latash, 2012). Despite the multiple degrees of freedom resulting from this abundance, as well as the variation across individuals, bipedal gaits that emerge from this system (i.e., walking and running) are remarkably similar. According to dynamical systems theory, these similarities in behavior emerge because of attractors (Kelso et al., 1981; Kelso, 2012). Specifically, limit cycle attractors may be primarily responsible for the convergence of joint motion to form the periodic behavior in gait (Ijspeert, 2008; Broscheid et al., 2018).

**Figure 1:**
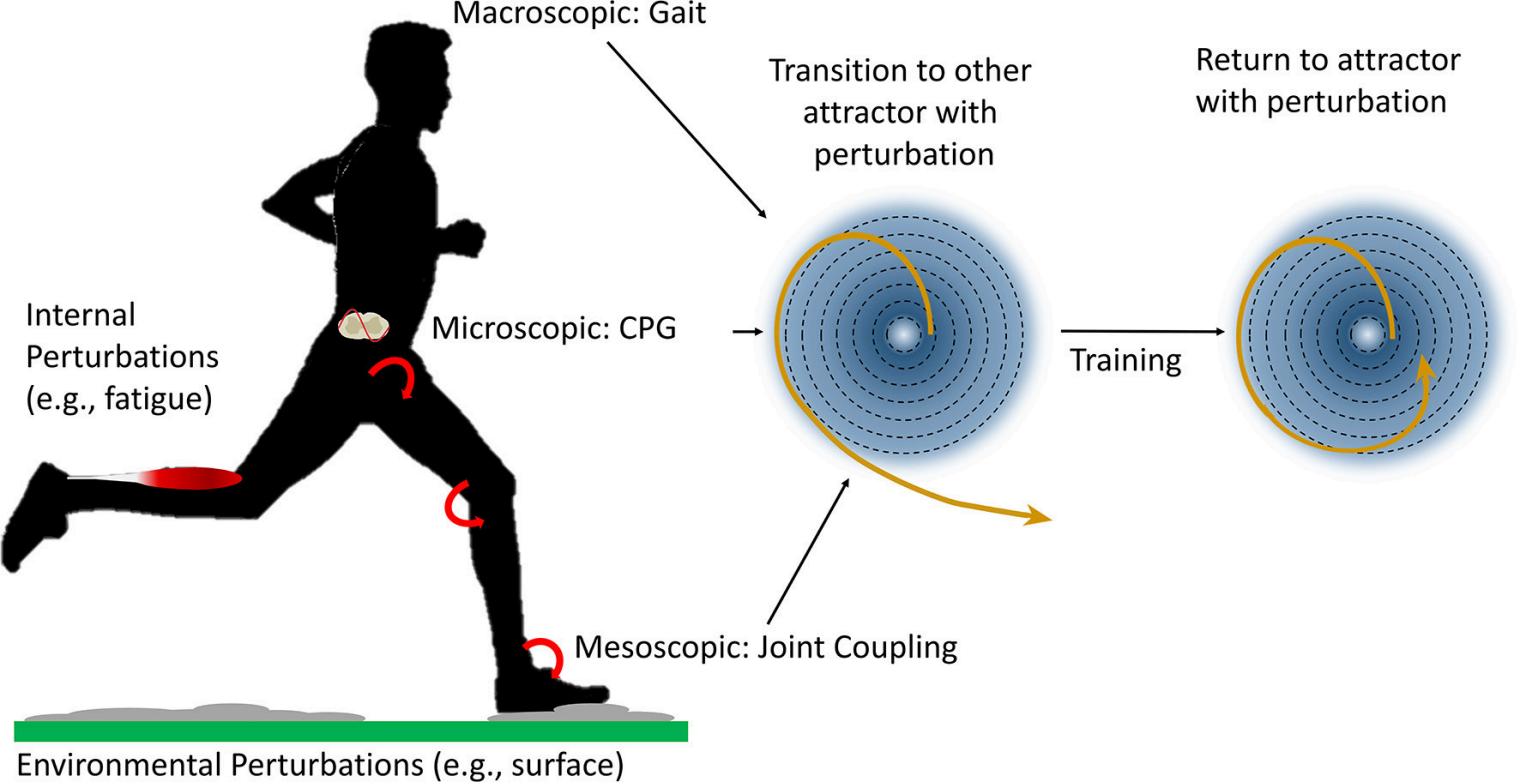
In bipedal gaits such as walking and running, gait, joint coupling and central pattern generators (CPG) may represent limit cycle attractors (coordination tendencies that cyclically repeat, simplified represented on the right in two dimensions based on Kitano, 2004) at a macroscopic, mesoscopic and microscopic level relative to each other when viewed from a top-down perspective, respectively. Internal and external perturbations from for example fatigue or an uneven surface can lead to a phase transition to another potentially less effective or less efficient (limit cycle) attractor. Large perturbations may for example lead to problems such as falls during walking or an ankle sprains while running. Training may increase the stability of attractors so that perturbations of a larger magnitude or perhaps greater frequency or unpredictability can be accommodated without a loss of stability.

Attractors represent coordination tendencies among system components (Davids et al., 2008), can be identified at multiple levels and emerge from the self-organization of the lower and higher-level components through circular causality (Haken, 1987). This means that the behavior of components at a higher level will be influenced (i.e., constrained) in a bottom-up manner by the behavior of components at the lower level and vice versa. With regards to locomotion, the two distinct human gaits, walking and running, represent two attractors at the macroscopic level (Diedrich and Warren, 1995; Lamoth et al., 2009), relative to the joint coupling of the ankle, knee and hip joints during these gaits that represents an attractor at a mesoscopic level (Diedrich and Warren, 1995), relative to the rhythmic neural activity of the central pattern generators that represents an attractor at a microscopic level (Cappellini et al., 2006; Ijspeert, 2008; Minassian et al., 2017) (Figure 1). Please note that we use micro-, meso- and macroscopic in relative terms, whereby the components at a mesoscopic level are macroscopic level components relative to the microscopic components.

Attractors in human locomotion may serve different purposes such as optimizing energetic and mechanical efficiency (Selinger et al., 2015; Kung et al., 2018), minimizing mechanical load (Kung et al., 2018), maintaining stability (Jordan et al., 2007) and increasing the robustness of the motion to perturbations resulting from internal (physiology) and external (environment) sources (Santuz et al., 2018). A decrease in stability of an attractor and an increased variability can induce a spontaneous phase transition to another attractor. An example of this in human locomotion is the walk to run transition, whereby a decreased stability of phase relationships of walking gait and increased variability in out-phase ankle-hip joint coupling with increasing walking speed results in a sudden self-organized transition around a speed of 2 m/s to the more stable running gait with a more in-phase joint coupling (Diedrich and Warren, 1995; Lamoth et al., 2009; Kung et al., 2018).

Other factors such as training (Zanone and Kelso, 1992; Kostrubiec et al., 2012), fatigue and aging (Stergiou and Decker, 2011) may also affect the magnitude of variability or the stability of the movement system and thereby induce a phase transition to a less optimal systems behavior. This may lead to reduced performance, injuries and pain. As effective locomotion is key to success in many sports, but also a necessity for functioning in daily life, understanding how the stability of the movement system in walking and running can be improved through training to enhance performance and reduce fatigue and injury would be beneficial. With the above points in mind, we propose that dynamical systems theory provides a framework for understanding locomotion and improving the effectiveness of interventions in locomotion-related problems. In this opinion article, we discuss two locomotor-related problems in populations of very different capacities, namely, falls among older adults and ankle sprain injuries in runners, and discuss how the application of dynamical systems theory can lead to novel approaches to intervention. Specifically, we hypothesize that applying small perturbations during locomotion may be effective for modifying the stability of specific locomotor attractors. Although using variability to improve performance is not novel [see studies on the variability of practice hypothesis (Schmidt, 1975), contextual interference effect (Magill and Hall, 1990) or differential learning (Beckmann and Schöllhorn, 2003; Schöllhorn et al., 2010; Serrien et al., 2018)], studies on these topics have mostly investigated discrete skills such as football kicking and shot-put and induced variability by practicing different movements rather than applying small unexpected perturbations during the actual movement to be improved. The use of small unexpected perturbations during movement may represent an effective way to further enhance performance.

## Dynamical Systems Theory and Ankle Sprain Injuries in Running

Running is a gait fundamental to many sports, but also an activity that is associated with a high injury incidence. A significant proportion of individuals participating in running or running-based sports sustain acute injuries such as ankle sprains (Van Gent et al., 2007; Fong et al., 2009). An ankle sprain can occur when high impact forces induce rapid inversion during ground contact, in particular when running on an irregular surface such as grass, sand or uneven sidewalks, or when changing direction. This inversion results in excessive stretch of the lateral ligaments that may lead to large strains or rupture (Fong et al., 2009).

Traditional approaches to ankle injury prevention and rehabilitation have applied training such as balancing on a wobble board or on one leg with the eyes closed (Schiftan et al., 2015). Although these approaches have been shown to be effective at preventing re-injuries in individuals with a history of ankle sprains, the evidence is less conclusive for ankle sprain prevention in individuals with no prior injury (Schiftan et al., 2015). Up to 70% of individuals with ankle sprain injuries report incomplete recovery and are therefore at a higher risk of re-injury (Anandacoomarasamy and Barnsley, 2005). One reason for the less conclusive evidence regarding primary prevention and incomplete recovery following an ankle sprain may be the different ways in which perturbations are corrected in traditional balance training and high-intensity movements such as running. According to the dynamical systems theory, a phase transition may occur from a combined reflex and preflex based correction of perturbations during tasks with no or minimum time pressure (e.g., traditional balance training on an unstable surface or slow walking) to a more preflex dominant correction in tasks with high time pressure such as the ground contact during high-speed running on uneven grass (Bosch, 2015). Reflexes may be strong and fast enough to correct smaller perturbations during traditional balance training, whereas they may be too slow and insufficient to prevent the effects of perturbations during (high-speed) running. Indeed, using a computational model of ankle inversion, DeMers et al. (2017) showed that reflexes took at least 80 ms to partly correct the perturbation, but failed to fully prevent excessive inversion. In contrast, moderate to high levels of co-activation were able to correct the perturbation within 60 ms due to the force-length-velocity properties of the muscle fiber and tendon elasticity, also known as preflexes (Loeb et al., 1999).

Applying small perturbations during running could potentially improve the robustness of running gait and in particular the motions of the ankle to perturbations by modifying the stability of the attractor via mechanisms such as alterations in step width and muscle activation at a mesoscopic level (Santuz et al., 2018). In the long-term, these acute mechanisms may translate into a more robust running gait pattern that is more prone to injuries via alteration in contact times and stride frequencies. We hypothesize that applying small perturbations during running may therefore be more effective for prevention and rehabilitation of ankle sprains compared to traditional balance training without time pressure, although further research is required to substantiate this notion. Also note that both approaches can complement each other and are not necessarily mutually exclusive.

## Dynamical Systems Theory and Falls Prevention in Older Adults

Walking is an essential gait for daily life but is also accompanied with an increased risk of falls with increasing age (Berg et al., 1997; Talbot et al., 2005). If we exclude environmental influences, we can address an individual's falls risk by looking either at the stability of their steady-state gait or at their behavior when they are brought out of balance to the extent that the locomotor behavior is altered. The latter has been the focus of much research, as many falls occur due to slipping or tripping (Berg et al., 1997; Talbot et al., 2005). Research on perturbation-based balance training has proliferated recently, during which the recovery reactions to sudden perturbations to balance or gait are trained (Mansfield et al., 2015; Okubo et al., 2016; Gerards et al., 2017; McCrum et al., 2017). However, as well as studying the reactive responses following balance loss, it is important to consider how the balance loss comes about, and if the robustness of the gait pattern to perturbations can be improved. In this context, rather than just using large perturbations that bring people out of balance, dynamical systems theory would suggest that applying smaller perturbations during gait that do not require a complete switch of locomotor behavior (phase transition in attractors) may also lead to positive improvements in gait stability via an increased robustness of the movement patterns. Both coping with small perturbations without a significant change in gait behavior and with large perturbations that do require some explicit recovery movements have previously been suggested as key requirements for stable gait (Bruijn et al., 2013), and both show significant declines with increasing age (Maki and Mcilroy, 2006; Süptitz et al., 2013; Terrier and Reynard, 2015; McCrum et al., 2016). The importance of studying stability during steady-state gait, in addition to reactive stability during larger perturbations, is supported by evidence of the relationship between decreased stability during steady-state gait and falls incidence (Hausdorff et al., 2001; Van Schooten et al., 2016; Bizovska et al., 2018).

Through the application of small perturbations during steady-state walking, the stability of specific locomotion attractors may be modified. One study has demonstrated alterations in motor primitives while walking and running on uneven, compared with even surfaces, creating activation patterns that were more robust to the perturbations (Santuz et al., 2018). If the basins of attraction of limit cycle attractors could be modified in older adults, this could mean that perturbations of a larger magnitude (or perhaps greater frequency or unpredictability) could be accommodated without significant loss in dynamic stability. For example, while walking over uneven ground, more frequent or larger undulations in the surface could be negotiated without loss of dynamic stability and without the need for subsequent large reactive balance corrections. One recent study had older participants walk on a treadmill with stable and unstable (water) loads in a backpack (Walsh et al., 2018). As would be expected, step variability was increased, and mediolateral dynamic stability decreased in the unstable load condition and electromyography activity was also increased to cope with the load (Walsh et al., 2018). If practiced over longer time periods, a more robust gait pattern may be the result via alterations such as step width or time, joint moments at the ankle to control center of mass velocity and muscle activation and motor primitives at a mesoscopic level. Further research is needed to examine the training effects of walking with small continuous unexpected perturbations and whether this translates to a more robust response to large perturbations and subsequently reduced falls risk, but such training represents one interesting avenue for future falls prevention interventions.

## Conclusion

Human locomotion can be conceptualized as a behavior of a dynamical system, with attractors that serve different purposes such as optimizing energetic and mechanical efficiency, minimizing mechanical load, maintaining stability and increasing the robustness of the motion to perturbations resulting from internal (physiology) and external (environment) sources. We have proposed that through the application of small perturbations during walking and running, the basin of attraction for specific locomotion attractors may be modified, which may have benefits for both maintaining gait stability and for reducing injury risk. Further research is required to elucidate the effectiveness of such interventions in different populations.

## Author Contributions

BVH and CM: Conception of the work; drafted the article. BVH: Prepared figure. All authors participated in discussions of the literature and concepts, reviewed and revised the article and approved the final version of the article.

### Conflict of Interest Statement

The authors declare that the research was conducted in the absence of any commercial or financial relationships that could be construed as a potential conflict of interest. The handling editor declared a shared affiliation, though no other collaboration, with one of the authors CM.
